# Enhanced MRI-based brain tumour classification with a novel Pix2pix generative adversarial network augmentation framework

**DOI:** 10.1093/braincomms/fcae372

**Published:** 2024-10-24

**Authors:** Efe Precious Onakpojeruo, Mubarak Taiwo Mustapha, Dilber Uzun Ozsahin, Ilker Ozsahin

**Affiliations:** Operational Research Centre in Healthcare, Near East University, Nicosia 99138, Turkey; Department of Biomedical Engineering, Near East University, Nicosia 99138, Turkey; Operational Research Centre in Healthcare, Near East University, Nicosia 99138, Turkey; Department of Biomedical Engineering, Near East University, Nicosia 99138, Turkey; Operational Research Centre in Healthcare, Near East University, Nicosia 99138, Turkey; Department of Medical Diagnostic Imaging, College of Health Sciences, University of Sharjah, Sharjah 27272, United Arab Emirates; Research Institute of Medical and Health Sciences, University of Sharjah, Sharjah 27272, United Arab Emirates; Operational Research Centre in Healthcare, Near East University, Nicosia 99138, Turkey; Department of Biomedical Engineering, Near East University, Nicosia 99138, Turkey; Department of Radiology, Weill Cornell Medicine, Brain Health Imaging Institute, New York, NY 10065, USA

**Keywords:** brain tumours, conditional deep convolutional neural network, generative adversarial networks, pix2Pix model, synthetic data

## Abstract

The scarcity of medical imaging datasets and privacy concerns pose significant challenges in artificial intelligence-based disease prediction. This poses major concerns to patient confidentiality as there are now tools capable of extracting patient information by merely analysing patient’s imaging data. To address this, we propose the use of synthetic data generated by generative adversarial networks as a solution. Our study pioneers the utilisation of a novel Pix2Pix generative adversarial network model, specifically the ‘image-to-image translation with conditional adversarial networks,’ to generate synthetic datasets for brain tumour classification. We focus on classifying four tumour types: glioma, meningioma, pituitary and healthy. We introduce a novel conditional deep convolutional neural network architecture, developed from convolutional neural network architectures, to process the pre-processed generated synthetic datasets and the original datasets obtained from the Kaggle repository. Our evaluation metrics demonstrate the conditional deep convolutional neural network model's high performance with synthetic images, achieving an accuracy of 86%. Comparative analysis with state-of-the-art models such as Residual Network50, Visual Geometry Group 16, Visual Geometry Group 19 and InceptionV3 highlights the superior performance of our conditional deep convolutional neural network model in brain tumour detection, diagnosis and classification. Our findings underscore the efficacy of our novel Pix2Pix generative adversarial network augmentation technique in creating synthetic datasets for accurate brain tumour classification, offering a promising avenue for improved disease prediction and treatment planning.

## Teaser

Onakpojeruo et al., propose using synthetic data generated by an advanced Pix2Pix model for brain-tumour classification, addressing data quality and patient privacy concerns. Their novel conditional deep convolutional neural network model achieved 86% accuracy, outperforming state-of-the-art models and demonstrating the efficacy of generative adversarial network augmentation in classification tasks.

## Graphical Abstract

**Graphical Abstract:**
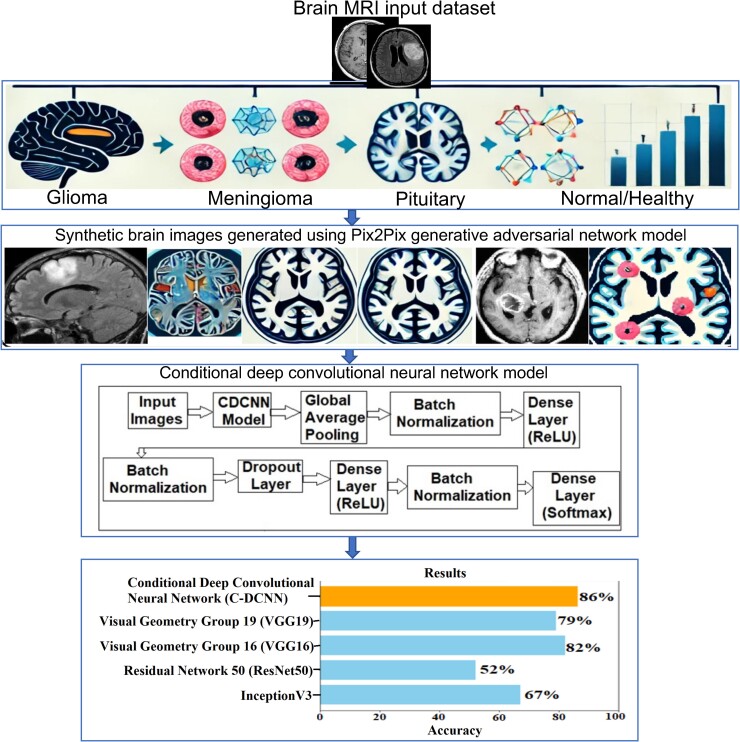

## Introduction

Brain tumours present a significant global health challenge, impacting individuals across all demographics.^1^ Cancer ranks as the second leading cause of death worldwide, with one in six deaths attributed to cancer-related complications.^2^ Among cancer types, brain tumours are particularly devastating due to their aggressive nature and low survival rates.^3^ The human brain, with its intricate network of ∼100 billion nerve cells, is a highly complex and delicate organ susceptible to tumour formation. These tumours can significantly alter brain function, posing serious health risks.^4,5^ To improve patient outcomes, brain tumours must be detected and classified early, according to whether they are malignant or benign.^6^ Benign tumours, confined to specific brain regions, are typically surgically treatable, while malignant tumours, with their propensity to spread to other body parts, present greater challenges.^7^ Roughly 700 000 people in the United States deal with brain tumour diagnosis, and just 36% of those people survive.^2^ Alarmingly, the incidence of brain tumours continues to rise, with tens of thousands of new cases diagnosed annually worldwide.^8^ Among the countless brain tumour types, glioma, pituitary and meningioma are the most prevalent, collectively constituting a significant portion of all diagnosed cases.^9^ Meningioma, a common benign tumour, originates in the brain and nervous central system surrounding membrane, while pituitary tumours primarily affect the pituitary gland. Gliomas, on the other hand, arise from brain tissue and are often malignant, underscoring the critical need for accurate tumour grading and classification to inform treatment strategies.^7-9^

**Figure 1:**
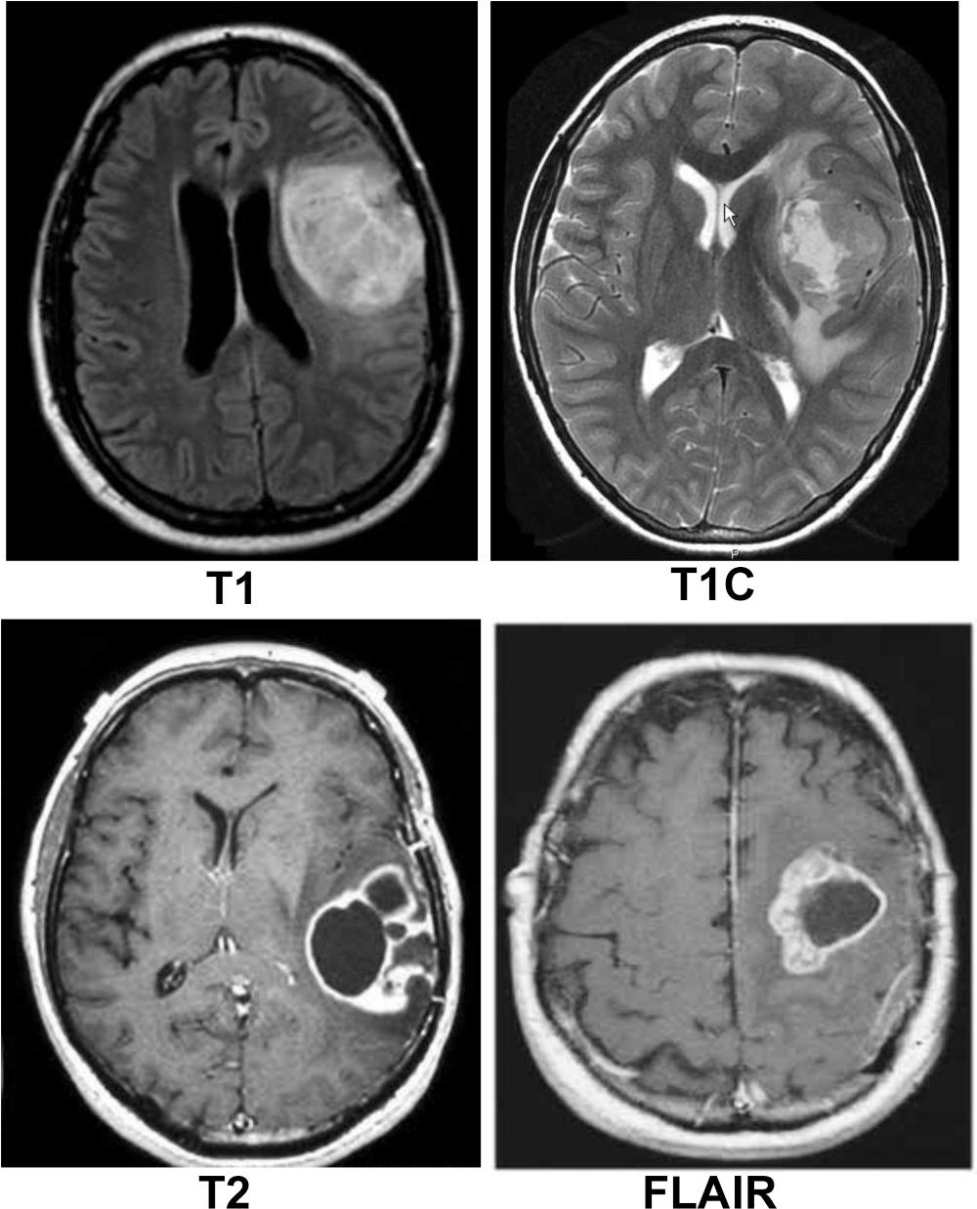
**Various MRI slices of the brain.** T1-weighted image (T1): Provides clear visualisation of the anatomy, showing high contrast between different tissues, particularly between grey and white matter. Used for identifying anatomical abnormalities of the brain; T1-weighted contrast-enhanced image (T1c): Similar to T1 images but with the addition of a contrast agent, usually gadolinium, which enhances the visibility of blood–brain barrier disruptions, tumours and other pathologies; T2-weighted image (T2): Highlights differences in the water content of tissues, making it useful for identifying oedema, inflammation and other conditions associated with increased water content, such as tumours or cysts. T2 images show fluid (like cerebrospinal fluid) as bright and white matter as dark; FLAIR: A special type of T2-weighted image where the signal from cerebrospinal fluid is suppressed, making it easier to see lesions near the ventricles and cortex, such as in cases of multiple sclerosis or brain tumours).

Brain tumour identification and diagnosis rely heavily on medical imaging techniques like computed tomography (CT) and magnetic resonance imaging (MRI).^10,11^ MRI simplifies the determination of the dimensions, form and precise position of abnormal tissue.^12^ Multiple MRI slices and protocols, including T1, T1c, T2 and fluid-attenuated inversion recovery (FLAIR), as illustrated in Fig. 1, are utilized for diagnosing brain malignancies,^13^ and utilized based on tissue properties.^14^ MRI, in particular, offers unparalleled imaging quality without subjecting patients to discomfort, making it the preferred imaging modality for brain tumour assessments.^15,16^ However, while manual analysis of MRI images is currently utilized for brain tumour detection and diagnosis, it is labour-intensive and prone to errors. Therefore, automated solutions like computer-aided diagnosis systems are essential to streamline this process and reduce the risk of inaccuracies. Despite advancements in automated segmentation techniques, challenges persist due to the scarcity of diverse, high-quality datasets and concerns regarding patient privacy.^14^

Recently, machine learning (ML) and deep learning (DL) techniques have become significant tools for the classification of brain tumours.^17^ DL, in particular, demonstrates superior performance in handling complex classification tasks compared to traditional ML approaches.^18^ Leveraging the latest advancements in DL technology. Despite the promise of DL-based solutions, the scarcity of diverse, high-quality datasets poses a significant challenge to model development. Concerns about privacy breaches and data leakage complicate dataset acquisition,^19,20^ as patients represented in data collected through CT or MRI scans may include identifiable images, such as head or facial images. This raises the risk that advanced DL models, like ‘Jekyll’, could accurately ascertain the identities of research participants.^21^ Traditional image augmentation techniques, while useful, are limited in their ability to generate diverse variations and safeguard patient privacy.^19-22^ In response, advanced techniques such as Pix2Pix generative adversarial network (GAN) augmentation offer a promising avenue for creating brain tumour synthetic datasets without compromising privacy or dataset integrity.^23-25^

### Related research

In recent years, numerous efforts have been made to develop accurate and efficient classification systems for brain tumours. These efforts have explored various methodologies, including traditional ML and DL techniques such as convolutional neural networks (CNNs) and transfer learning. Existing literature predominantly focuses on binary classification, which is relatively straightforward due to the discernible shape of tumours. However, the challenge intensifies when classifying brain tumours into multiple categories due to their high degree of similarity. Traditional ML methods have been used by several authors, the method often involves sequential stages and manual feature extraction, utilising techniques such as discrete wavelet transform (DWT),^26-28^ grey level co-occurrence matrix^26,29^ and genetic algorithms.^30,31^ Support vector machines (SVMs)^26,32^ are commonly employed for classification due to their efficacy, while other methods such as random forest, extreme learning machines and sequential minimal optimisation have also been explored.^32-35^ It is noticeable that there is manual feature extraction in the traditional ML method, and it is both time-consuming and prone to errors. Conventional ML techniques depend on manually designed functions that need accurate initial data, like the tumour’s location, and are prone to human errors. Hence, it is essential to create a strong and efficient system that does not rely on manual features. The DL technique has been extensively utilized in medical imaging and brain tumour categorisation. DL does not rely on manually created features; however, pre-processing steps and selecting the appropriate architecture may be needed to enhance classification accuracy.^18^ In the domain of DL, CNNs have emerged as a prominent tool for brain tumour classification using MRI.^1,32^ Researchers have utilized brain datasets like Figshare, created by Cheng,^36^ to develop efficient classification methods.^37-39^ employed DWT and Bag-of-Words models for feature extraction and achieved a classification accuracy of 100% using SVM. Additionally, CNN-based approaches, often incorporating transfer learning, have demonstrated remarkable performance. For instance,^40^ utilized the GoogleNet model with transfer learning to achieve a classification accuracy of 97% on the Figshare dataset using SVM.

Recent advancements in deep transfer learning have revolutionized brain tumour detection and classification, offering a lifeline for thousands of individuals worldwide. A study by Ullah *et al*.^41^ explores the efficacy of nine pre-trained transfer learning classifiers, including Inceptionresnetv2, Xception and Resnet50, in identifying brain tumours. Utilising a fine-grained classification approach on the Figshare dataset, the inceptionresnetv2 TL algorithm emerges as the top performer, achieving impressive accuracy rates of 98.91%. Further validation against hybrid approaches, combining CNN for feature extraction and SVM for classification, reaffirms the superiority of transfer learning algorithms in accurately classifying brain MRI images. Various studies have explored diverse DL architectures and transfer learning techniques for brain tumour classification. These include the utilisation of models such as InceptionV3, ResNet-50, VGG-16 and VGG-19, with notable achievements in accuracy ranging from 91% to 99%.^42-46^

Additionally, the use of data augmentation and methods such as Faster region-based CNN (Faster R-CNN)^47^ have enhanced classification accuracy. Image augmentation is crucial for improving model performance and reducing the requirement for manual image collection and labelling.^48^ It accomplishes this by algorithmically creating and increasing datasets. There are two main types of image augmentation techniques: basic or traditional methods and augmentation methods based on DL algorithms.^49^ Image augmentation methodology involves transforming an image dataset using geometric or colour processing methods.^50^ On the other hand, augmentation using DL algorithms depends on DL techniques.^49^ In supervised ML and DL pipelines, the original dataset is initially divided into distinct sets for training, validation and testing purposes, or just for training and testing. Image augmentation is often performed on training (and maybe validation) images during model training and optimisation. Test data is left un-augmented to prevent data leakage. However, image augmentation can still be used during testing to evaluate model performance.^12^ Traditional image augmentation approaches used in developing models have limitations in generating varied variations due to simple geometric and colour alterations.^51^ Moreover, there is a potential for inadvertent data breaches and inadequate privacy protection for patients included in the data. This is especially worrisome when the data include images of the head, faces, or similar depictions that might easily and accurately disclose the identities of research participants.^19-22^ Beyond traditional data augmentation techniques, Vanilla GAN, which was introduced in 2014 by Garcea *et al*.^52^ in the field of computer vision, offers innovative and promising approaches to representation learning. This could increase the diversity and improve the original datasets, leading to the creation of more realistic samples. The samples do not include any identifying personal data, but they do maintain the statistical characteristics of the original data. Since 2017, there has been an increase in research focused on using GANs to enhance the performance of models in classification tasks by augmenting or synthesising brain tumour images.^53^ These include the utilisation of GAN models such as DCGAN, Pix2Pix GAN, CycleGAN, TumorGAN, AGGrGAN and WGAN, etc. with notable achievements in accuracy ranging from 80% to 99%.^23-25,54-60^ GANs are acknowledged as the most effective techniques for identifying and obtaining MRI and CT image features in segmentation and classification tasks.^53^ The improvements indicated are mostly due to GANs effectively increasing the probability density of the data-generating distribution by using density ratio estimation in an indirect way of supervision. Additionally, GANs can uncover the high dimensional latent distribution of data, leading to notable enhancements in the extraction of visual features.^53^

The application of Pix2Pix GAN for the generation of synthetic data in medical research represents a novel approach that remains underexplored in the current literature. In the context of brain tumour image classification, the application of Pix2Pix GAN for generating synthetic datasets has not been extensively researched. Although there have been applications of GANs in medical image synthesis for brain tumour classification in studies,^23-25,54-56^ the specific use of Pix2Pix GAN for generating synthetic brain tumour data to enhance model performance is still largely unexamined. The proposed study presents a new DL model, the conditional deep convolutional neural network (CDCNN), aimed at improving the accuracy of brain tumour classification through MRI images. The CDCNN model seeks to surpass current state-of-the-art models in brain tumour detection and diagnosis through the integration of a novel Pix2Pix GAN augmentation and various pre-processing techniques. This study aims to enhance brain tumour classification and improve patient care outcomes through detailed performance analysis and comparative evaluations. This study aims to determine the most effective DL framework for the precise classification of brain cancer into four categories: normal, glioma, meningioma and pituitary tumours. In contrast to earlier studies that concentrated on binary classification and conventional augmentation methods, this research examines the intricacies of multiclass classification, specifically in differentiating between brain tumour types through the application of the Pix2Pix GAN augmentation technique. The research employed the innovative CDCNN model and conducted a comparative analysis of its performance against transfer learning architectures such as Residual Network50 (ResNet50), Visual Geometry Group 16 (VGG16), Visual Geometry Group 19 (VGG19) and InceptionV3. The study utilizes multiple pre-processing techniques and integrates a deep dense block to improve the model’s classification performance. We aim to provide valuable insights into brain tumour classification and enhance the development of more accurate diagnostic tools through rigorous experimentation and comprehensive analyses.

## Materials and methods

**Figure 2:**
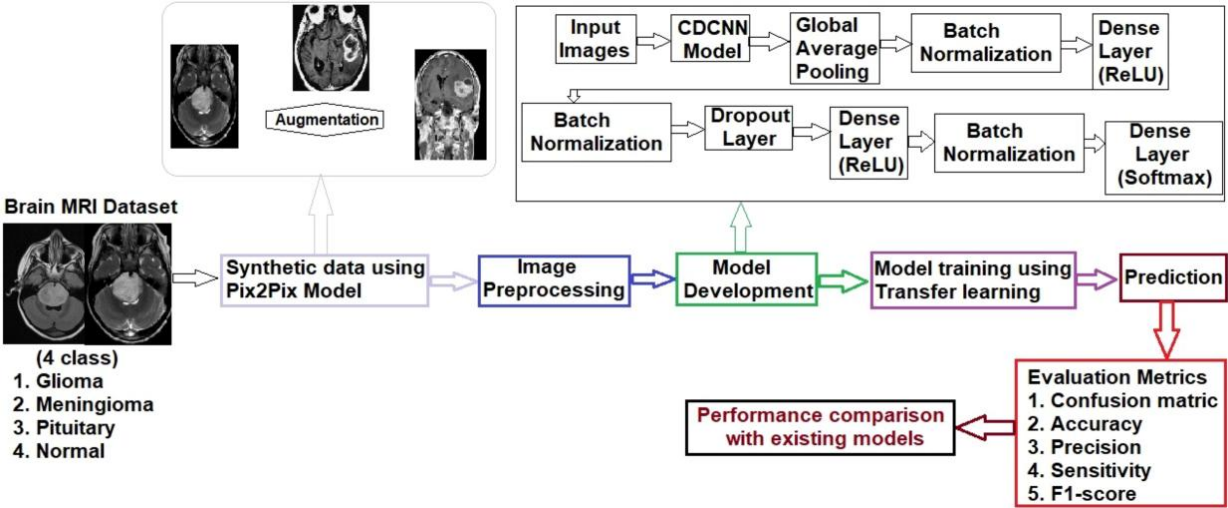
Experimental design and flow of the study.

Figure 2 displays the proposed method for classifying brain tumour disease using our innovative CDCNN deep transfer learning model.

### Data collection

**Figure 3:**
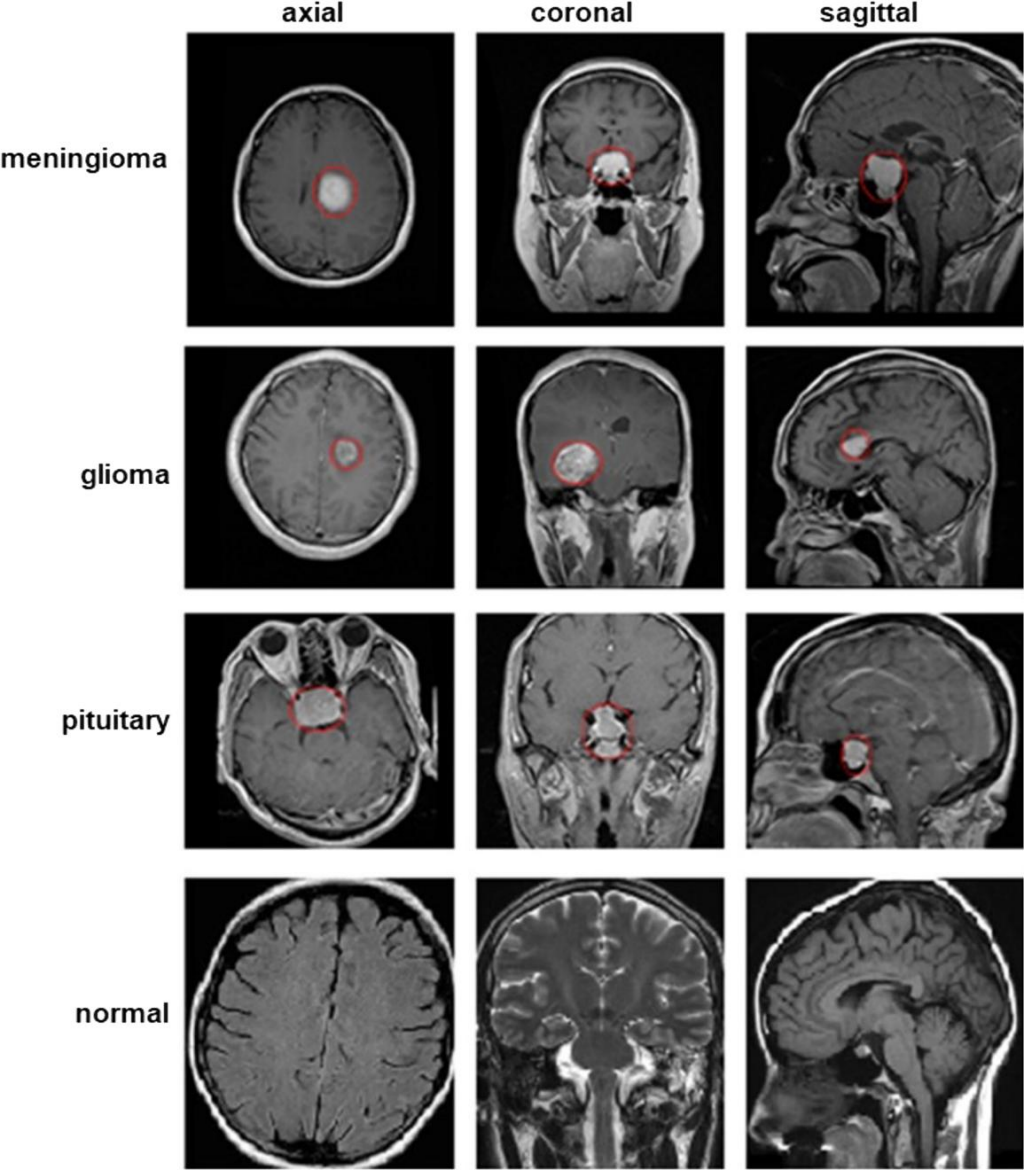
**A class-labelled brain tumour dataset of a brain MRI image.** ^61^ This figure showcases examples from the Brain Tumour Classification dataset, consisting of MRI slices categorized into four classes: normal, glioma, meningioma and pituitary. The tumours in the glioma, meningioma and pituitary images are delineated by a red outline, highlighting the regions of interest within the brain MRI images.

Recent models frequently employ the Figshare benchmark brain tumour dataset^36^ or the Brain Tumor Classification dataset^61^ from the Kaggle open-source data repository for performance evaluation. This study employs the brain tumour classification dataset to evaluate the efficacy and robustness of the proposed method. The brain tumour classification dataset were sourced from the Kaggle open-source data repository.^61^ The dataset includes 3264 brain MRI slices from male and female patients, divided into four categories: normal (500 images), glioma (926 images), meningioma (937 images) and pituitary (901 images). Figure 3 presents brain MR images from four distinct class datasets. The images depict the tumour outlined in red.

### Creating synthetic datasets using our novel Pix2Pix GAN model

Pix2Pix is a GAN architecture created for image-to-image translation tasks.^62^ The concept was presented by researchers at the University of California, Berkeleyin 2017.^63^ Pix2Pix is renowned for its ability to generate realistic images by learning the mapping between input images and corresponding output images in a supervised manner.^63^ Pix2Pix comprises a generator network and a discriminator network, both trained concurrently in an adversarial manner. The generator processes an input image from a source domain (e.g. a black-and-white sketch) to convert it into a related output image in a target domain (e.g. a colourful rendition of the sketch). The discriminator's role is to differentiate between authentic images from the target domain and fake ones created by the generator.^62,63^ The generator in training tries to reduce the disparity between its generated images and the authentic images from the target domain, while the discriminator strives to amplify this difference. The adversarial training process results in the generator learning to create output images that are indistinguishable from genuine images, as determined by the discriminator.^64^ Pix2Pix GAN utilizes a pixel-wise loss function, like L1 mean absolute error (MAE) or L2 loss, with the adversarial loss to promote pixel-level precision in the generated images. Pix2Pix GAN excels in its ability to handle a wide range of image-to-image translation tasks such as colourisation, style transfer, semantic segmentation and edge-to-image translation. The flexibility is attained by conditioning the generator and discriminator networks on the input images within a conditional GAN framework, where the input image acts as a conditioning variable.^25^

The architecture used in this study is based on the Pix2Pix GAN model, which is a conditional generative adversarial network (CGAN) consisting of a generator and a discriminator. The generator maps input images (*x*) and random noise (*z*) to target images (*y*), while the discriminator distinguishes between real and fake images. The following equation represents Pix2Pix’s objective function:


(1) $$L_{Pix2Pix}(G,\;D)=E_{x,yP\;data(x,\;y)}[\log D(x,\;y)]+\;E_{xPdata(x),\;zPz(z)\;}[\mathrm{loglog}\;(1-D\;(x,\;G(x,\;z)))\;]$$

**Figure 4:**
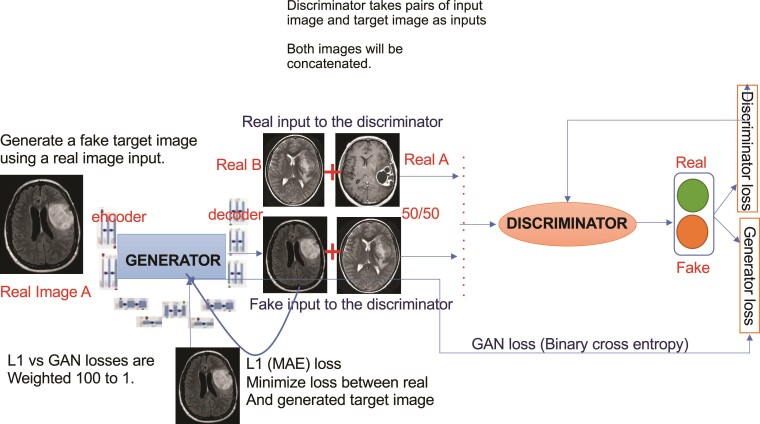
**Flowchart of image-to-image transmission with Pix2Pix GAN.** This figure illustrates the image-to-image translation process using the Pix2Pix GAN model. The flowchart depicts the interaction between the generator and discriminator networks during training. The generator transforms input images into target domain images, while the discriminator attempts to differentiate between real target images and those generated by the generator. The adversarial training process allows the generator to produce highly realistic images that closely resemble the target domain.

Unlike traditional GANs, Pix2Pix GAN employs a PatchGAN discriminator that operates on image pairs rather than single images.^25^ The training process of the Pix2Pix GAN involves iterating over a set number of training iterations. Within each iteration, the model undergoes multiple steps where mini-batches of samples from the data distribution and noise prior are sampled. The discriminator is updated by ascending its stochastic gradient using a loss function that compares real and fake images, while the generator is updated by descending its stochastic gradient based on the discriminator's feedback as shown in Fig. 4.

**Figure 5:**
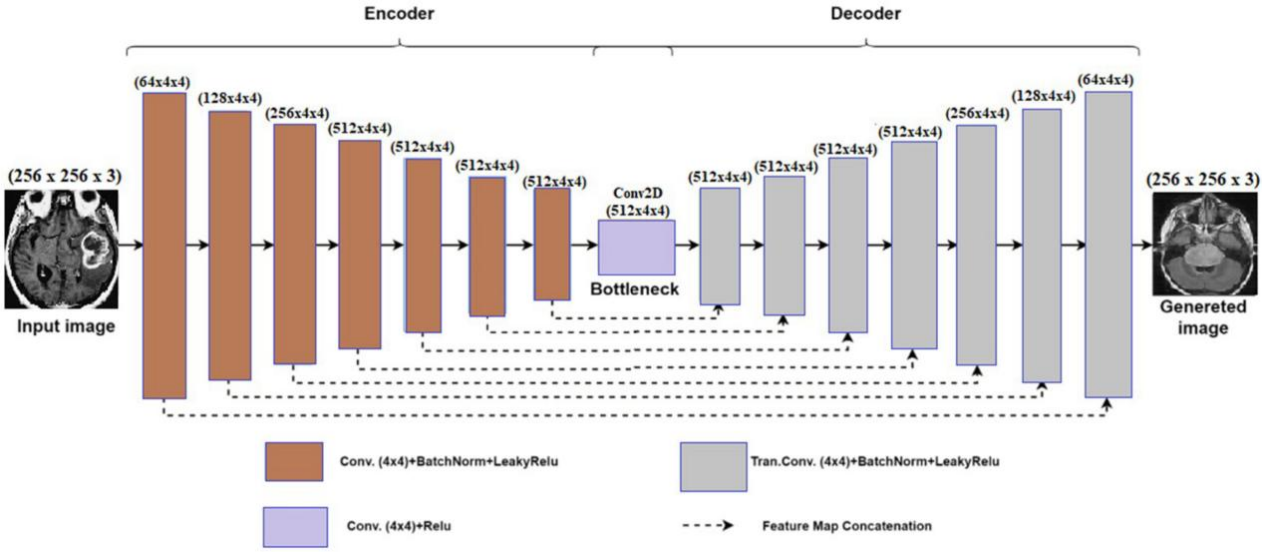
**Architecture of the generator.** This figure illustrates the architecture of the generator in the enhanced Pix2Pix model, inspired by the U-Net architecture. The generator comprises an encoder and a decoder, arranged in a ‘U’ shape. The encoder processes the input image through convolutional and pooling operations, generating a feature vector. The decoder then upsamples the feature vector using upconvolutions, concatenating the upsampled feature maps with corresponding feature maps from the encoder. This structure allows for precise image generation, particularly useful in tasks requiring high accuracy in synthetic image creation.

**Figure 6:**
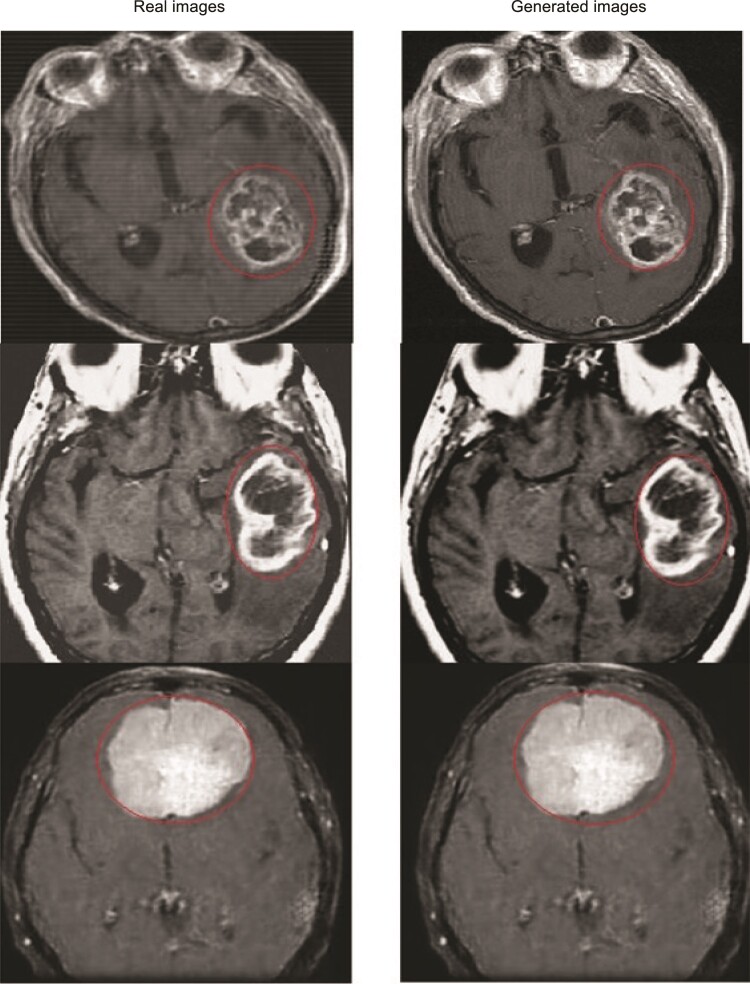
**Real versus generated images using Pix2Pix GAN model.** The tumour is delineated by a red outline. This figure compares real images with images generated by the Pix2Pix GAN model. The tumours are delineated by a red outline, highlighting the areas of interest. The comparison demonstrates the effectiveness of the Pix2Pix GAN model in generating synthetic images that closely resemble the real images, particularly in accurately representing the tumour regions.

We developed a generator inspired by the U-Net architecture to improve the performance of the Pix2Pix model for generating synthetic images. The U-Net architecture comprises an encoder and a decoder, forming a shape analogous to the letter ‘U.’ The encoder transforms input images into a feature vector through convolutional and pooling operations, whereas the decoder employs upconvolutions to rescale the feature vector to the original input image dimensions. The upsampled feature map in the decoder's upconvolution layer is concatenated with feature information from each convolution layer of the encoder during the upsampling process. Consequently, U-Net generates a more accurate segmentation map.^25^ The encoder and decoder layers were augmented, and hyperparameters including kernel dimensions, stride size and padding were modified accordingly. Figure 5 illustrates the proposed generator architecture, while Fig. 6 presents a comparison between real images and images generated by the Pix2Pix GAN model.

### Image pre-processing

Image pre-processing improves essential and complex features. All algorithms are influenced by noise; therefore, appropriately pre-processed images can enhance classification tasks. Image pre-processing techniques are categorized according to the intended pixel region size. These techniques function on the adjacent pixels of sub-images to reduce noise and distortion, thereby enhancing image quality. Low image quality, external factors and a limited user interface can compromise MRI images, leading to diminished visual information and challenges in processing. The datasets were pre-processed at various stages to ensure optimal accuracy prior to conversion into the proposed structure.

#### Enhancing image contrast

Image contrast enhancement techniques are used to improve the visibility of certain areas in brain tumour images. This enhances the image quality and makes significant features more distinguishable.

#### Conversion from PNG to JPG

Conversion from PNG to JPG is necessary because the generated MRI images of brain tumours are initially in PNG format, while the datasets obtained from the Kaggle repository are in JPG format, which can cause compatibility issues with CNN frameworks. The PNG images are converted to JPG format to improve data administration, reduce file size and increase compatibility with CNN frameworks. Libraries like Pillow (Python Imaging Library, PIL), OpenCV (Open Source Computer Vision Library) and Matplotlib are used for this conversion procedure.

#### Greyscale conversion and rescaling

The greyscale images are adjusted and standardized to maintain consistency in data representation among various images. The resized and standardized images are stored as JPG files for additional analysis.

#### Cropping to eliminate unwanted areas

Numerous brain MRI images generated may have unnecessary space and noise that could impede the model's performance. The images are cropped to eliminate unnecessary areas, directing attention exclusively to the centre section of the brain.

#### Standardising image dimensions

All the generated images have 256 × 256 dimensions, on the other hand the downloaded primary data from Kaggle have different dimensions ranging from 200 to 652. Standardising MR images from the dataset to a uniform size is crucial to ensure consistent model input due to their different sizes. The resize function is used to standardize the shape of all brain tumour images suitable for the architectures utilized in the study.

#### Data partitioning

The image data is divided into three subsets: training, testing and validation, based on the Pareto principle with an 80–20 split ratio. 80% of the images are used for training and validation, and the remaining 20% are set aside for testing. This partitioning approach guarantees an equitable distribution of data to enhance the training and evaluation of the CDCNN model. Table 1 displays the dataset details and the distribution of data for training and testing the model.

**Table 1:** Distribution of the datasets

References	Glioma	Meningioma	Pituitary	Normal	Total images	Train/test dataset
Kim et al.^61^	926	937	901	500	3264	80/20
Synthetic datasets by Pix2Pix GAN model application						
	2500	2500	2500	2500	10 000	80/20

### Application of CDCNN model

**Figure 7:**
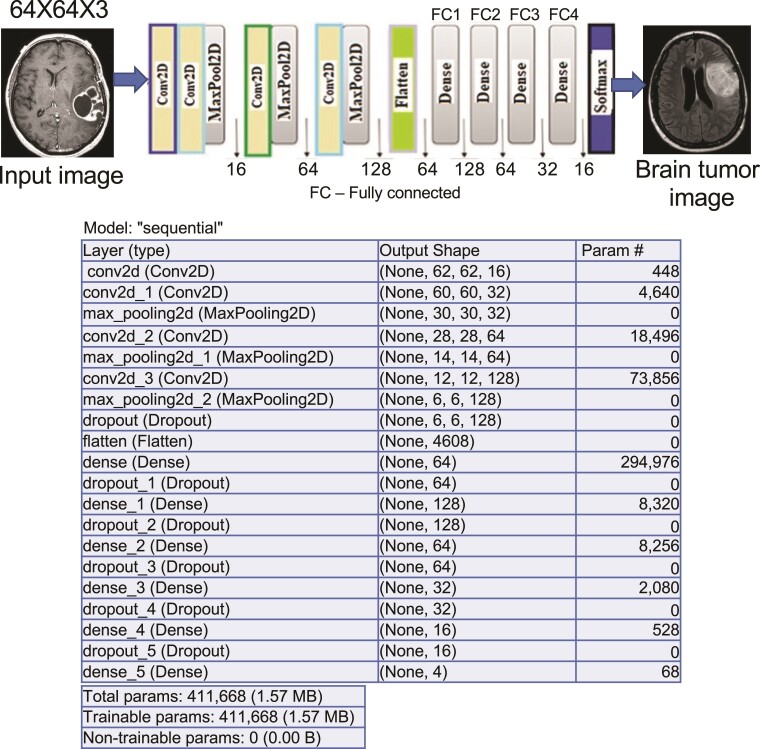
**Architecture of the proposed CDCNN model.** This figure illustrates the architecture of the proposed CDCNN model. The model is designed for image classification tasks, featuring multiple convolutional layers, max-pooling layers and fully connected layers. The architecture incorporates dropout for regularisation and uses the SoftMax activation function for the final classification layer. The model’s unique components, including attention mechanisms, residual connections and optimized hyperparameters, contribute to its enhanced performance in handling complex image classification and object recognition tasks.

The CDCNN represents an innovative CNN model that employs conditional multi-modal contextual fusion for the extraction of distinctive features from the dataset. The study utilized a specialized CDCNN model adapted to our generated datasets to illustrate the efficacy of a CNN model specifically designed and optimized for this research, incorporating distinct modifications to the data to attain optimal performance. This is consistent with the data employed and the framework of the problem we seek to resolve. The CDCNN model was initially developed as a basic CNN model subsequently enhanced through the addition of layers and the adjustment of hyperparameters to optimize performance. During hyperparameter tuning, measures were implemented to prevent overfitting and underfitting, thereby ensuring sufficient generalisation to unknown data. The model's performance is comparable to that of the most advanced pre-trained state-of-the-art models assessed in this study. The model is engineered to effectively manage image classification and object recognition tasks. The framework comprises modules intended for feature extraction, object detection via the region proposal network and subsequent classification. A CNN model was developed with a clearly defined architecture employing the classification detection method. The model consists of multiple convolutional and dense layers, incorporating dropout for regularisation, to extract output information from an individual image. The architecture of our CNN incorporates a two-dimensional CNN. The architecture consists of four convolutional layers and three max-pooling layers. Two layers utilized kernel sizes of 3 × 3 and 2 × 2 for pooling operations. The classification utilized a SoftMax activation function along with four fully connected layers. The architecture comprised layers with 128, 64, 32 and 16 neurons, as illustrated in Fig. 7. The SoftMax activation function is utilized for classifying images into their corresponding categories. The SoftMax function converts output values into a probability distribution by normalising them to a range between 0 and 1. The equation below defines the SoftMax activation function:


(2) $$\mathrm{SoftMax}\;{\left(x\right)}_{i}=\frac{\;{exp\;}^{x}i}{\sum_{j=1}^{k}\;e^{x}j}$$

#### Training information

The Adam optimizer, utilising gradients, was applied with a batch size of 32. A dropout rate of 25% was applied to the convolutional and fully connected layers. The categorical_crossentropy loss function was employed to evaluate the alignment between predicted probabilities and true class outputs, ensuring that the model's outputs are compared with one-hot encoded labels for each class, thus facilitating multiclass classification tasks. We assemble the model using accuracy criteria. All layers of the network utilized the ReLU activation function, with the exception of the final layer, which employed the SoftMax activation function. Our model is characterized by a unique and innovative approach across multiple dimensions. It includes auxiliary components such as batch normalisation layers, dropout layers, residual connections and attention mechanisms. The modifications improve training stability, prevent overfitting, optimize gradient flow and enhance the model's ability to capture long-range dependencies. The model is optimized for the efficient execution of image classification tasks. Additionally, it is applicable for object recognition tasks within a cohesive and integrated framework. This design is both adaptable and effective, eliminating the necessity for distinct models for different tasks. The incorporation of an attention mechanism in our model enhances the representation of relationships among various spatial locations. This enables the model to concentrate on pertinent regions within the image and comprehend contextual relationships. This attention technique significantly improves performance in image classification tasks. Convolutional layers, when integrated with residual connections, enhance the model's ability to effectively learn intricate characteristics and structures of images. Convolutional layers extract hierarchical features, whereas residual connections enhance gradient flow and support the training of more intricate models. The model serves as a significant resource for researchers and practitioners due to its adaptability and versatility.

### Optimisation and hyperparameter tuning

Hyperparameters play a critical role in model performance and training, necessitating adjustment, particularly for our novel CDCNN model based on CNN architectures. Hyperparameters are predefined settings selected by the researcher, rather than being acquired by the model during training. The model's capacity, regularisation and convergence speed are significantly affected by these factors.^65^ We maintained the hyperparameters of established models such as ResNet50, VGG16, VGG19 and InceptionV3 for the classification task in our study. Opting for this choice guarantees the retention of knowledge gained through transfer learning, while alterations to these hyperparameters can markedly affect the model's structure and performance. The grid-search optimisation technique was utilized for the CDCNN model in the classification task. The optimal model configuration for enhanced performance is established by systematically exploring a predefined set of hyperparameter combinations through grid-search optimisation techniques.^66^ This strategy is essential for assessing the model's performance across different combinations through a systematic exploration of the hyperparameter space. Optimal configuration for a specific task can be identified by evaluating various hyperparameter values and selecting the one that produces the best results. Four primary hyperparameters were tuned: learning rate, batch size, epochs and optimizer selection. The batch size specifies the quantity of data gathered prior to weight updates in the model, typically varying from 10 to 100. The number of epochs, between 30 and 100, determines how often the model processes the entire dataset during training. Seven optimizers were considered: SGD, RMSProp, Adagrad, Adadelta, Adam, Adamax and Nadam, to investigate various optimisation methodologies. Multiple learning rates (0.0001, 0.001, 0.01, 0.1 and 0.2) were evaluated to assess their effects on the model's performance and convergence. The optimal hyperparameters for the CDCNN model were identified as 32 batches, 50 epochs, the Adam optimizer and a learning rate of 0.0001 through grid-search optimisation. The parameters were chosen to optimize the model's performance for the specific task while mitigating issues like slow convergence and overfitting.

### Summary of the hyperparameter selection for Pix2Pix GAN and CDCNN models

#### Learning rate

We tested several learning rates (0.0001, 0.001, 0.01, 0.1 and 0.2) to observe their impact on the model's performance and convergence. The optimal learning rate was determined to be 0.0001 for our CDCNN model.

#### Batch size

The batch size determines the number of data collected before updating the weights in the model, ranging from 10 to 100. We chose a batch size of 32 for optimal performance.

#### Number of epochs

The number of epochs, ranging from 30 to 100, dictates the frequency at which the entire dataset is processed by the model during training. We selected 50 epochs for training.

#### Optimizer selection

We considered seven optimizers: SGD, RMSProp, Adagrad, Adadelta, Adam, Adamax and Nadam to explore different optimisation methodologies. The Adam optimizer was chosen for its balance of speed and performance.

#### Dropout rate

To prevent overfitting, a dropout rate of 25% was implemented on the convolutional and fully connected layers.

#### Loss function

The categorical_crossentropy loss function was employed to evaluate the alignment between predicted probabilities and actual class outputs, facilitating a comparison of the model's outputs with one-hot encoded labels for each class, thereby rendering it appropriate for multiclass classification tasks.

### Architecture of the CDCNN model

Figure 7 shows the schematic of our proposed CDCNN model. With the use of dropout regularisation and many convolutional and dense layers, the network is optimized to efficiently process image classification and object recognition tasks. An overview of the design and settings is provided here:

#### Conv2D layers

The model includes three convolutional layers with varying filter sizes and activation functions to capture different levels of image features.

#### MaxPooling2D layers

Max-pooling layers are employed after each convolutional layer to downsample the spatial dimensions of the feature maps.

#### Dropout layers

Dropout layers are added to prevent overfitting by randomly dropping units during the training process.

#### Flatten layer

This layer flattens the input, creating a single long feature vector.

#### Dense layers

Several dense layers are used for the final classification, with a SoftMax activation function in the output layer to classify the images into their respective categories.

### Statistical analysis

To assess the quality of synthetic images produced by the Pix2Pix GAN, we further computed the mean squared error (MSE) between the original and synthetic images. The MSE calculates the average of the squared differences between the original and synthetic images. It serves as a quantitative evaluation of image quality and offers insights into the success of the Pix2Pix GAN in generating high-quality synthetic data. To determine the MSE between the original and synthetic images, we carried out the following procedures:

Step 1: Perform image pre-processing to standardize the format and size of the images.

Step 2: Calculate the differences between each pixel in the original and synthetic images.

Step 3: Calculate the square of each difference.

Step 4: Calculate the average of the squared deviations.

The MSE formula is defined as follows:


$$\mathrm{MSE}=\frac{1}{N}\;\overset{N}{\underset{i=1\;}{\sum }}\;{\left(Ii-Si\right)}^{2}$$

where *Ii* is the pixel value of the original image, *Si* is the pixel value of the synthetic image and *n* is the total number of pixels when the implementation of the MSE was computed in the Python environment using NumPy version: 1.24.3.

#### Computational results

The MSE was calculated for a subset of both real and artificially generated images taken from the brain tumour datasets. The pixel values ranged from 0 to 255. The average MSE was calculated to be 0.056, suggesting a close resemblance between the synthetic and real images. The results of this study provide evidence for the efficacy of the Pix2Pix GAN in producing synthetic data of superior quality, which may be utilized for training and evaluating models.

## Results

An 11th Gen Intel(R) Core (TM) i7–11700KF @ 3.60 GHz processor, along with TensorFlow and Python, was used to develop the model on a Windows 10 Pro edition, version 22H2, 64-bit operating system. A learning rate of 0.0001 and a batch size of 64 were used in the 200 iterations of the Adam optimizer during the training procedure. Every image, both input and output, was downsized to 256 × 256. Using the TensorFlow library, we defined the generator and discriminator models, and a step function was used to execute the training. Improved image generation is achieved by integrating parts of PatchGAN, U-Net and Vanilla GAN loss into the model architecture. Both the synthetic and augmented brain images were produced by our innovative Pix2Pix GAN algorithm. Figure 6 shows both the original and the generated images. Both the primary datasets obtained from Kaggle and the synthetic dataset created using the Keras package and Python within the Jupyter Notebook environment were used for model training and evaluation in the study. A total of 3264 real datasets and 10 000 synthetic datasets were used to create the pre-processed data. Of this, 80% was set aside for training and 20% for testing. Afterward, the innovative CDCNN model was trained using them. A randomly selected 20% sample of the training data was used to validate the model, which allowed us to evaluate its performance. After that, we trained the model for 50 iterations. Following the third max pooling layer and on top of the thick layers, the dropout regularisation method was applied. When it comes to regularising data, dropout regularisation is straightforward and easy to implement. By selectively turning off neurons during training, it is possible to develop a neural network that is both simple and efficient. One way to lessen the likelihood of overfitting is to use a simple neural network. We added two callbacks to make training better, make the model work better and reduce overfitting. The initial callback, called EarlyStopping, checks the validation loss and stops training before a certain number of epochs have passed if the loss doesn't improve. Reducing the validation loss beyond a certain number of epochs causes the learning rate to be decreased in the second callback, ReduceLROnPlateau. A patience value of 3 is used to apply this reduction. The codes we used for this work are openly available at https://github.com/Efep3332/GANs-Generated-Synthetic-Dataset.

### Validation metrics

Developing, testing and deploying ML models relies heavily on performance evaluation metrics. Better and more efficient AI solutions can be more easily developed with their help.^67^ Model evaluation offers a way to objectively assess the model's sensitivity, specificity, accuracy and precision, among other performance indicators.^68^ In order to compare different models and assess how well they function, performance evaluation metrics are necessary. A key component in making DL models reliable is evaluating their performance metrics, which in turn improves their transparency and interpretability. On both the synthetic and primary datasets, the new CDCNN model's prediction efficacy was evaluated using the performance assessment metrics. According to the results of both datasets, the CDCNN model was quite accurate in detecting and classifying brain malignancies as either glioma, meningioma, pituitary or normal. According to Table 2, the main datasets achieved a 72% accuracy rate; however, the synthetic dataset that was created achieved 86% accuracy. Based on the generated datasets, the model appears to be capable of correctly categorising brain tumours into four groups in the vast majority of cases, with extraordinary precision scores of 83% for glioma tumours, 88% for meningioma tumours, 81% for pituitary tumours and 94% for no tumours. The model correctly detected all positive and negative cases, as shown by the recall scores of 70%, 82%, 96% and 98% for the four classes of the synthetic datasets. The model's accuracy in classifying the four types of brain tumours in MRI scans is indicative of its responsiveness to synthetic datasets created artificially and its therapeutic usefulness. This provides strong evidence that the model is highly specific and can accurately detect tumour absence. Further, the model's capacity to achieve 76%, 85%, 88% and 96% F1 scores for the four classes on the created synthetic datasets shows that it successfully balances sensitivity and precision. When F1 is high, it means that recall and precision are well-balanced. Consequently, the model successfully reduced the occurrence of false positive results while reliably detecting four kinds of brain tumours. The model's 86% accuracy score for synthetic datasets and lower accuracy for primary/original datasets obtained from Kaggle indicate that it performs better on generated synthetic datasets than on real datasets when it comes to accurately classifying synthetic MRI images into their respective brain tumour classes. The substantial benefits of synthetic datasets, such as increasing the size of the accessible main datasets and reducing data privacy concerns, are further supported by the findings, which provide support to their use in classification tasks. When it comes to accurately detecting and classifying brain tumours in MRI images, the CDCNN model is really outstanding. This phenomenon has important therapeutic implications since it allows for more accurate and faster diagnosis, which is especially helpful in areas with limited access to medical professionals and resources. As a powerful tool for identifying and classifying brain tumours from MRI images, the CDCNN model shows encouraging possibilities.

**Table 2:** Classification report of the novel CDCNN model

Type of dataset	Class data	Precision %	Recall %	f1-score %	Accuracy %
Generated synthetic datasets	Glioma tumours	0.83	0.70	0.76	86
	Meningioma tumours	0.88	0.82	0.85	
	Pituitary tumours	0.81	0.96	0.88	
	No tumours	0.94	0.98	0.96	
Primary/original datasets	Glioma tumours	0.70	0.72	0.71	72
	Meningioma tumours	0.70	0.56	0.62	
	Pituitary tumours	0.65	0.74	0.69	
	No tumours	0.79	0.90	0.84	

### Confusion matrix

**Figure 8:**
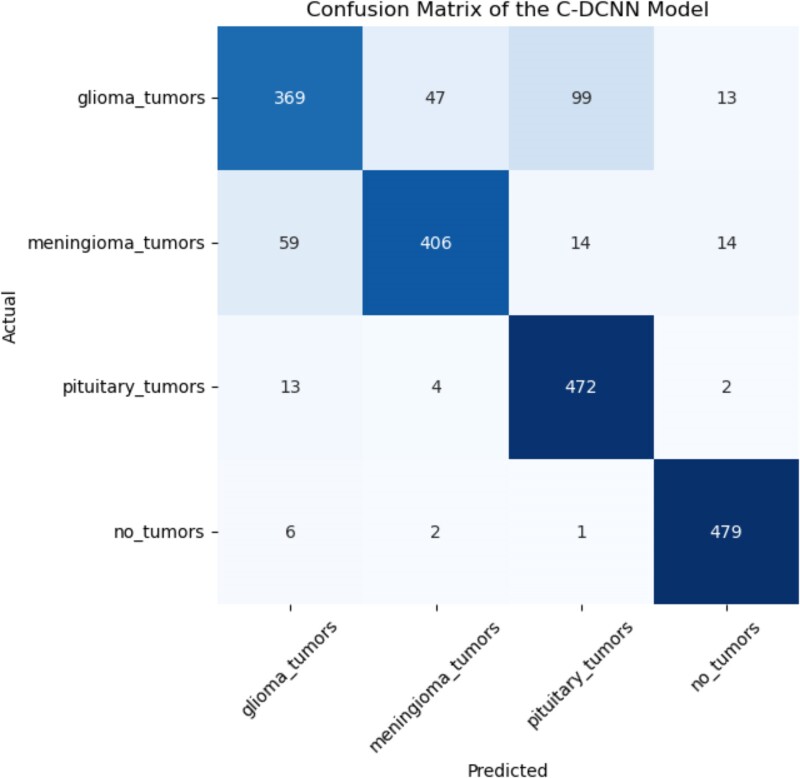
**Confusion matrix of the CDCNN model.** This figure displays the confusion matrix for the CDCNN model, illustrating its performance in classifying brain MRI images into four distinct classes: glioma, meningioma, pituitary and normal. The matrix provides a detailed breakdown of true positive (TP), true negative (TN), false positive (FP) and false negative (FN) predictions. The highest classification accuracy was observed for normal images (479 correctly classified), followed by pituitary (472), meningioma (406) and glioma (369). The overall accuracy achieved by the model is 86%, demonstrating its effectiveness in accurately identifying tumour presence across the four categories.

When assessing the performance of models, statisticians frequently use the confusion matrix. Using the confusion matrix, we can evaluate how well our model classifies every image into four distinct categories. The predicted quantities of rue positive (TP), true negative (TN), false positive (FP) and false negative (FN), are displayed in the matrix. Confusion matrices are essential for assessing models’ accuracy and efficacy because they offer a concise visual depiction of the model's performance.^69^ In order to increase the accuracy and reliability of diagnoses, it is necessary to perform a thorough analysis of the matrix in order to identify the model's strengths and weaknesses. The confusion matrix clearly shows that the CDCNN model produced very good results. The results shown in Fig. 8 show that our novel approach successfully categorizes patients into four groups: glioma, meningioma, pituitary and normal. The model shows a pituitary tumour image ratio of 472 images, a meningioma image ratio of 406 and a glioma image ratio of 369, with 479 being the highest. According to the results of the confusion matrix analysis, the results obtained from the test dataset are considered adequate. The proposed model properly identified 1726 instances while misclassifying 274 cases, according to the examination of 2000 test images. Overall, the model's accuracy rate of 86% was satisfactory, and it produced satisfactory results. All four classes will be accurately classified according to this result. This model allows for the detection of brain tumours in real time.

### Learning curve

**Figure 9:**
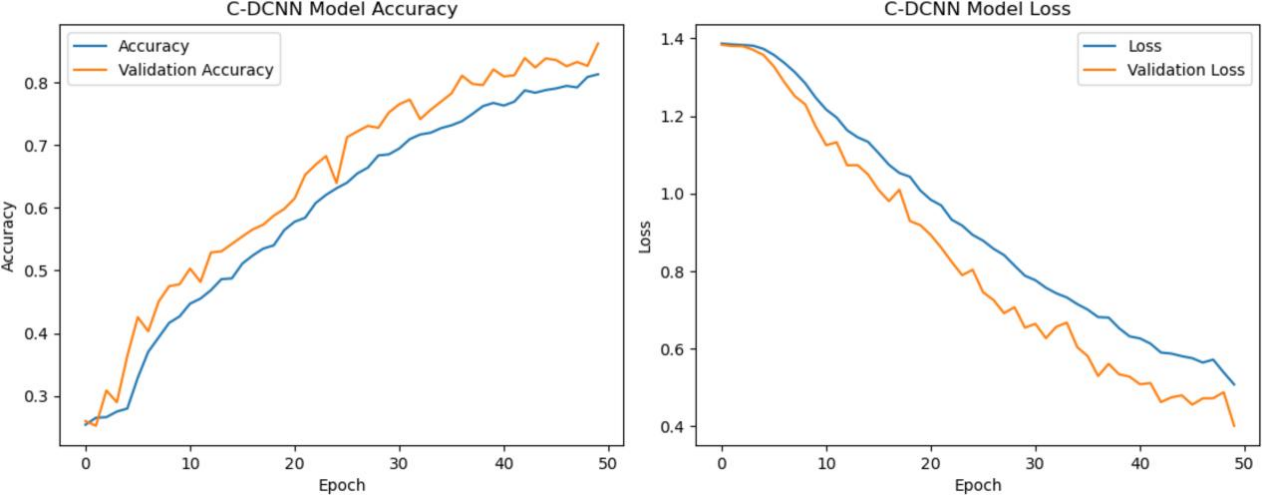
**Accuracy and loss of CDCNN model for generated synthetic datasets.** This figure illustrates the learning curves of the CDCNN model over 50 epochs. The accuracy curve shows the model’s progression in training and validation accuracy, while the loss curve demonstrates the reduction in training and validation loss as the model learns. The CDCNN model exhibits steady improvement in accuracy and a consistent decline in loss, eventually stabilising at an accuracy of 86.30% and a validation loss of 0.37037. These curves highlight the model’s robust fit to the synthetic datasets without signs of overfitting or underfitting, indicating effective learning and generalisation.

When evaluating the performance of models, learning curves are crucial. It sheds light on a model's consistency and accuracy throughout training, which helps to spot any performance problems and gives direction for making the model better. Over time, the model's accuracy on both the training and validation datasets will evolve, as shown by the model accuracy learning curve. Depending on the situation with the training data, the evaluation system can determine if the model is overfitting or underfitting. An overfitted model is highly accurate when trained on one dataset but significantly less accurate when tested on another, suggesting that better generalisation is needed to deal with novel and unfamiliar data. When a model is underfit, it fails to properly detect patterns in the data, as seen by its poor accuracy on both the training and validation sets. By keeping an eye on the model's learning curve, one may determine the ideal amount of training epochs to avoid overfitting and underfitting. The CDCNN model successfully captured the underlying patterns in the generated data without experiencing overfitting or underfitting, demonstrating a robust and dependable fit. As the epochs progress, the training accuracy steadily improves, showing that the model is constantly learning and improving. Figure 9 shows that training began slowly at epoch 0 but steadily improved with time. At half of the epochs, the synthetic dataset had a learning rate of 1.0000e-05, an accuracy of 86% during validation, a loss of 40% during training and a training loss of 51%. Verifying the model's congruence with the training data requires testing it on the validation data, which consists of novel and unexplored information. The reliability of the test is given equal weight in the assessment. The validation accuracy follows a similar pattern to the training accuracy, showing a slow improvement with some minor variance. Nevertheless, the test's accuracy rises steadily until it hits its limit at epoch 50 for synthetic datasets. With a test loss of 0.37037, the accuracy has reached 86.30%. Based on these results, it seems unlikely that adding more training will help the model perform better on fresh data. The learning curve for model loss shows how a model's loss function changes throughout the course of training, across several epochs. As the model becomes more in line with the data, the training loss gradually decreases, indicating that it is making progress. Starting at 1.3869 in epoch 1, the training loss for synthetic datasets dropped to 0.5109 in epoch 50. This finding suggests that the model becomes better at predicting the target outcome with less room for error. Meanwhile, validation loss showed a consistent decrease from epochs 1–13, then variations from epochs 14–29, and finally a stable state at epoch 30. The validation loss began to steadily decline at epoch 30 and continued to do so until it stabilized at epoch 50. On a dataset that was not utilized for training but was utilized to assess the model's efficacy, the validation loss quantifies the discrepancy between the predicted and observed results. The result is that the model's performance is evaluated using previously unseen data. When the model's training and validation losses have balanced out after many epochs, we say that the model is well-fit. Overfitting occurs when the validation loss is high and the training loss is low, whereas underfitting may occur when the training loss and validation losses are both large. The learning curves of the model's accuracy and loss must be carefully examined in order to assess the model's performance and determine the necessary adjustments.

### Receiver operating characteristic (ROC) curve

**Figure 10:**
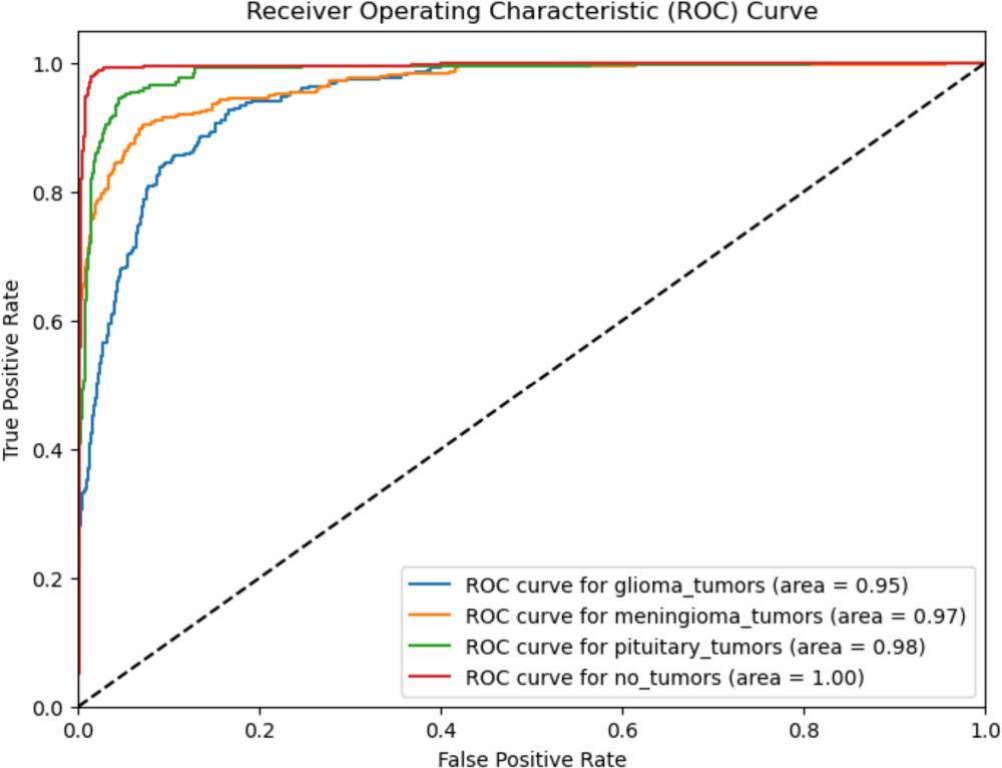
**ROC curve for CDCNN model for both synthetic.** This figure presents the ROC curves for the CDCNN model, illustrating its performance in classifying brain MRI images into four distinct categories: glioma, meningioma, pituitary and normal. The AUC values are 0.94715 for glioma, 0.96831 for meningioma, 0.98451 for pituitary and 0.99549 for normal, indicating the model's high diagnostic accuracy across all categories. The ROC curve demonstrates the model's ability to balance sensitivity and specificity, with higher AUC values reflecting superior performance in distinguishing between true positive and false positive cases.

The assessment metric known as the area under the ROC curve (AUC ROC) holds significant importance in the context of classification tasks. It offers a visual depiction of the diagnostic accuracy of a classification model. This metric offers a thorough assessment of a classifier's effectiveness in meeting all possible classification criteria. AUC ROC assesses the sensitivity (rate of correctly identified positive cases) and specificity (% of correctly identified negative cases). The metric provides a concise numerical representation of the classifier's ability to categorize brain tumours into four distinct classifications. A higher AUC ROC value signifies improved performance of the model. The ROC curve is a visual depiction of the performance of a classification model as the threshold for differentiation is modified. In contrast, the AUC is a quantitative metric utilized to evaluate the overall efficacy of a classification model, relying on its ROC curve. The AUC value quantifies the model's overall capacity to differentiate between distinct classes, with a value of 1 indicating perfect classification and 0.5 indicating random variation.^70^ Figure 10 displays the AUC ROC curves for four unique patient classes: glioma (0.94715), meningioma (0.96831), pituitary (0.98451) and normal (0.99549). These curves demonstrate the robust classification capability of the CDCNN model in effectively categorising brain tumours into their various classes. This suggests that the model possesses the capacity to effectively differentiate between genuine positive instances (MRI images that are correctly identified) and false positive cases (MRI images that are incorrectly classified) with a significant level of accuracy. The model exhibits exceptional efficacy in precisely diagnosing brain tumours, as indicated by its elevated AUC value. These findings suggest that the CDCNN model can provide reliable and accurate diagnostic support for oncologists.

## Discussion

### Comparative evaluation of the CDCNN model with state-of-the-art models

Our novel CDCNN model is compared to existing advanced DL models such as ResNet50, VGG16, VGG19 and InceptionV3. This allows us to establish a benchmark for evaluation, assess its capacity to generalize, contribute to the advancement of the field, and aid in making decisions that are applicable in the real world. These transfer learning models are widely recognized and commonly used in the field of computer vision. They demonstrate outstanding performance and have made significant contributions to tasks like image recognition and classification.^71,72^ The performance, competitiveness and prospective superiority of the CDCNN model can be accurately evaluated by a comparison analysis with established models. The purpose of this comparison is to position the distinctive CDCNN model in relation to existing state-of-the-art techniques and verify its reliability and importance in the domain of computer vision. The implementation of advanced transfer learning models, such as the CDCNN model, was carried out using Jupyter Notebooks. We employed a fine-tuning technique in our advanced CDCNN model to address the problems of overfitting and underfitting. This process was carried out utilising all the cutting-edge models included in our comparative evaluation.

The ResNet50 architecture is widely recognized for its utilisation of a deep residual learning approach. This approach addresses the problem of diminishing gradients in intricate networks, facilitating the training of exceptionally deep models. The model has demonstrated efficacy in numerous image classification tasks and is highly acknowledged for its ability to effectively capture complex features from CT and MRI images. The VGG16 and VGG19 were developed by the Visual Geometry Group (VGG) at the University of Oxford as CNN models. These models demonstrate a coherent architecture, characterized by the presence of many layered convolutional and fully connected layers. VGG16 and VGG19 have gained significant recognition for their exceptional and distinctive performance in the domain of large-scale image categorisation tasks. These models have exhibited remarkable accuracy due to their profound and intricate feature extraction capabilities. The concept of inception modules, also known as GoogleNet, was introduced by InceptionV3. These modules employ parallel convolutions at different spatial resolutions to effectively capture multiscale information. The proposed architectural design aims to reduce computational complexity while maintaining a level of accuracy that is considered high. The utilisation of InceptionV3 has been widely employed in numerous image recognition applications, demonstrating remarkable performance in the domains of object detection and localisation.

**Figure 11:**
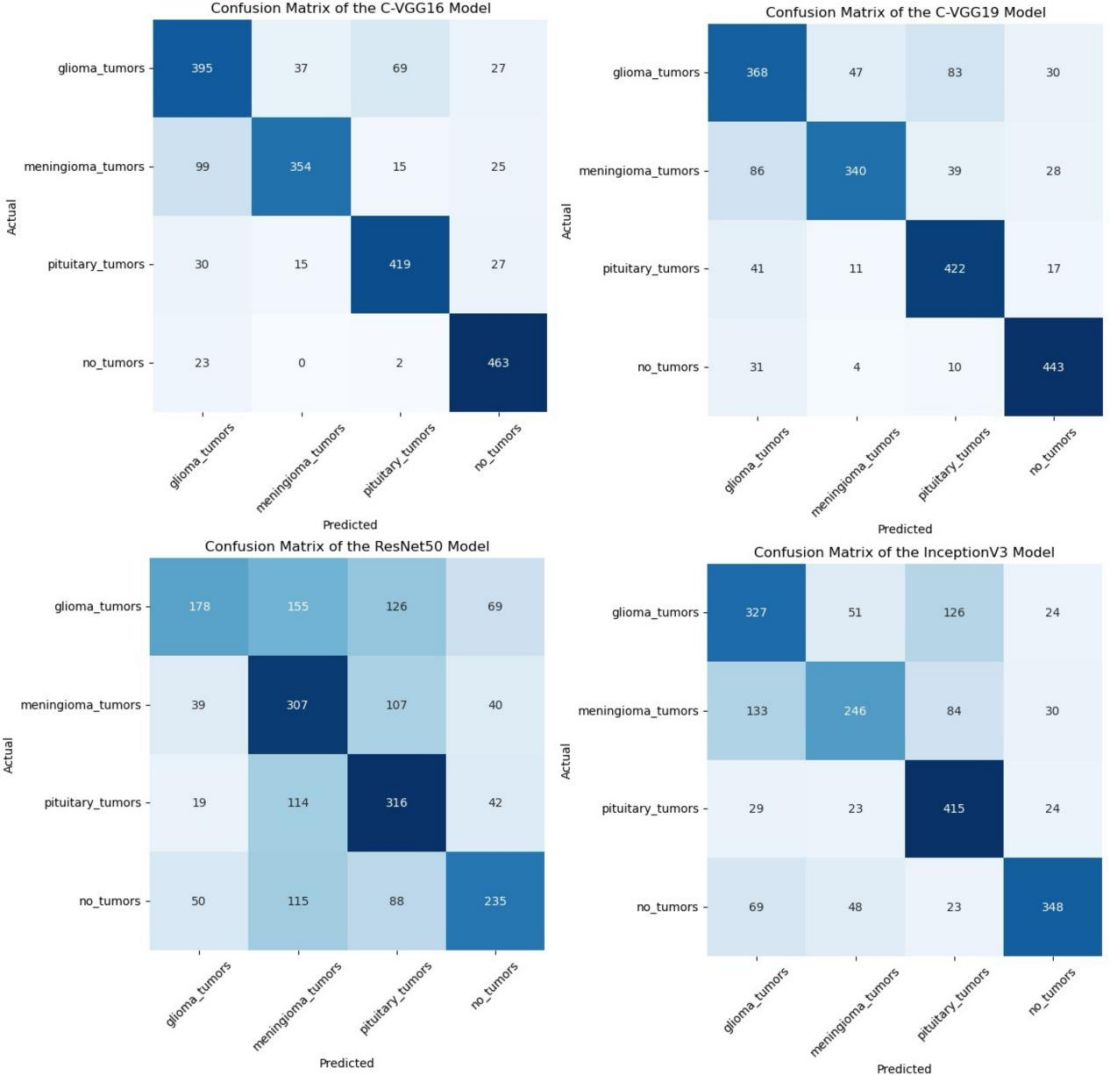
**Confusion matrix for the four state-of-the-art models.** This figure presents the confusion matrices for four advanced DL models; VGG19, VGG16, ResNet50 and InceptionV3 used in the classification of brain MRI images into four distinct classes: glioma, meningioma, pituitary and no tumour. The confusion matrix visually depicts the model's performance by showing the true positives, true negatives, false positives and false negatives for each class. The comparative analysis of these models against the novel CDCNN model demonstrates varying levels of accuracy and classification effectiveness, with the CDCNN model showing superior performance across all categories.

The performance of the novel CDCNN model is compared to state-of-the-art advanced DL models, revealing notable disparities and presenting significant therapeutic implications. The performance of the CDCNN model was found to be superior in comparison to other models, as it displayed enhanced predictive capabilities. In terms of accuracy, precision, sensitivity, specificity, F1 score, AUC and loss, the CDCNN model demonstrates superior performance compared to VGG16, InceptionV3, VGG19 and ResNet50. When trained on the generated synthetic datasets, the model achieves an accuracy of 86%, surpassing the performance of the other models, as shown in Table 3. The precision, recall and f1-score of the CDCNN model demonstrate higher performance in comparison to ResNet50, VGG16, VGG19 and InceptionV3. The F1 score, which is outstanding, exhibits a remarkable balance between precision and sensitivity. The confusion matrix is the main tool for evaluating errors in classification tasks. We have constructed a confusion matrix for the four state-of-the-art models as shown in Fig. 11. Furthermore, the CDCNN model exhibits a perfect minimum AUC value of 0.94 across all four classes, showcasing its remarkable ability to differentiate between different classes. Moreover, it demonstrates a diminished loss value, indicating enhanced optimisation and a decreased occurrence of errors. The improved accuracy of the CDCNN model holds significant clinical significance. The classification accuracy of the CDCNN model in classifying brain tumours in MRI scans into four separate classes (glioma, meningioma, pituitary and no tumour) is 86%, indicating a high level of reliability and accuracy in the results. This extraordinary level of accuracy can greatly aid oncologists and healthcare professionals involved in the diagnosis and treatment of malignant tumours. The implementation of this approach reduces the probability of misdiagnosis, enabling timely detection and appropriate intervention, ultimately improving patient outcomes.

**Table 3:** Comparative evaluation of the CDCNN model with state-of-the-art models

Type of dataset	Models	Class data	Precision %	Recall %	f1-score %	Accuracy %
Generated synthetic datasets	CDCNN	Glioma tumours	0.83	0.70	0.76	86
		Meningioma tumours	0.88	0.82	0.85	
		Pituitary tumours	0.81	0.96	0.88	
		No tumours	0.94	0.98	0.96	
	VGG19	Glioma tumours	0.70	0.70	0.70	79
		Meningioma tumours	0.85	0.69	0.76	
		Pituitary tumours	0.76	0.86	0.81	
		No tumours	0.86	0.91	0.88	
	VGG16	Glioma tumours	0.72	0.75	0.73	82
		Meningioma tumours	0.87	0.72	0.79	
		Pituitary tumours	0.83	0.85	0.84	
		No tumours	0.85	0.95	0.90	
	ResNet50	Glioma tumours	0.62	0.34	0.44	52
		Meningioma tumours	0.44	0.62	0.52	
		Pituitary tumours	0.50	0.64	0.56	
		No tumours	0.61	0.48	0.54	
	InceptionV3	Glioma tumours	0.59	0.62	0.60	67
		Meningioma tumours	0.67	0.50	0.57	
		Pituitary tumours	0.64	0.85	0.73	
		No tumours	0.82	0.71	0.76	

The remarkable effectiveness of the novel CDCNN model can be attributed to several factors, such as its unique architectural design, efficient training approach and improved ability to capture and represent the relevant features in brain tumour MRI images. The CDCNN model demonstrated enhanced efficiency in capturing and extracting relevant features for brain tumour classification through the incorporation of specific design elements, namely the mixed-scale dense convolution layer, self-attention mechanism, hierarchical feature fusion and attention-based contextual information. The CDCNN model was constructed using an optimum configuration and effective training procedures, which involved careful identification of hyperparameters such as learning rate, batch size and regularisation techniques. The use of these combinations will accelerate the convergence process and augment the model's capacity to uncover a more ideal answer.

Among the four considered advanced DL models, ResNet50, which is a highly intricate model known for its deep architecture and skip connections, exhibited the least favourable performance.^73^ ResNet50 demonstrated an accuracy of 52% with the synthetic data and 54% with the original datasets. These results reveal that ResNet50 exhibited a comparatively higher incidence of both false positives and false negatives, resulting in worse predictive outcomes. This result may occur due to the unique characteristics of the used data and the complex architecture of the ResNet50 model. The InceptionV3, alternatively referred to as the GoogleNet model, demonstrated somewhat inferior performance in comparison to the VGG19, an improved iteration of the VGG16 model. The InceptionV3 attained an accuracy of 67% with the synthetic data, and 51% with the original datasets, VGG19 attained an accuracy of 79% with the synthetic datasets, and 72% with the original datasets, and VGG16 attained an accuracy of 82% with the synthetic datasets, and 70% with the original datasets. The decreased precision, recall, F1 score, AUC, and accuracy of the model are apparent. The observed disparity may be attributed to factors such as the increased complexity of the InceptionV3 and VGG19 models, leading to an insufficient representation of the specific characteristics relevant to the classification tasks.

In the context of the broader research landscape, several related methods have been proposed to enhance GAN improvements and ensure privacy-preserving data analysis in the medical field. For instance, the generative adversarial U-net for domain-free few-shot medical diagnosis proposed in a study by Chen *et al*.^74^ employs a U-Net architecture with a GAN framework to enable domain-free medical diagnosis, thereby improving the generalisation capability of models across different medical datasets. Similarly, another study by Han *et al.*^75^ discussed the privacy-preserving multisource domain adaptation for medical data to address the challenge of maintaining patient privacy while enabling multisource domain adaptation for medical data. These methods provide valuable context and demonstrate the relevance of our work within the broader scope of medical data analysis and synthetic data generation.

## Conclusion

This study presents a method that highlights the DL model's medical significance in detecting, diagnosing and classifying brain tumours using MRI images produced by the Pix2Pix GAN model and images taken from the original datasets obtained from the Kaggle repository. The method specifically employs the novel CDCNN model. We evaluated the efficacy of cutting-edge DL models trained on both real and synthetic datasets, including ResNet50, VGG16, VGG19 and InceptionV3. The potential of the CDCNN model, which was developed from CNN architectures, as an effective tool for rapid and accurate diagnosis is highlighted by its outstanding accuracy, precision, sensitivity, specificity and F1 score. Brain tumours may be easily categorized into four different groups using the new CDCNN model. Using synthetic datasets generated by the Pix2Pix GAN model to train DL models can achieve equivalent performance to primary and augmented datasets, according to the key discovery of this study. Research employing synthetic datasets generated by GANs has shown better accuracy, which is in line with previous findings.^23-25,54-56,76-78^ This proves that synthetic datasets have a lot of promise as a well-established DL augmentation method for practical use in industry. Typically, these applications make use of datasets of a very small size and provide a multitude of benefits, including the protection of users’ privacy. Reducing the time and opportunity cost of obtaining real-world data is a major benefit of the proposed Pix2Pix GAN technique for producing synthetic data. The findings of this study contribute to the ongoing advancements in AI-powered cancer diagnostics and demonstrate how DL models can improve diagnostic accuracy and patient care, even when fed synthetic data.

### Implications and contributions to the field

Our study demonstrates that the proposed CDCNN model significantly improves the diagnostic accuracy of brain tumour classification. By integrating multimodal data and leveraging advanced GAN architectures for synthetic data generation, we achieve higher precision in identifying and categorising different types of brain tumours. This advancement is critical for early diagnosis and treatment planning, potentially improving patient outcomes. The model's performance across different datasets indicates its robustness and generalizability. Utilising the Brain Tumor Classification dataset from Kaggle, along with synthetic datasets generated by the Pix2Pix GAN model, ensures that the model can handle diverse imaging conditions and tumour types. This robustness makes the model applicable to various clinical settings, providing reliable diagnostic support regardless of the specific imaging equipment or protocols used. Implementing the CDCNN model in real-time clinical workflows can enhance radiologists’ efficiency and accuracy. The model's ability to process and classify brain MRI images rapidly can assist radiologists in identifying tumours more quickly, reducing the time required for diagnosis and allowing for more timely interventions. Furthermore, the interpretability methods developed in this study, such as visualising the regions of MRI images that contribute most to the classification, provide valuable insights for medical professionals, supporting their decision-making process. The innovative use of the Pix2Pix GAN model for creating synthetic datasets addresses the common challenge of limited annotated medical images for training AI models. By generating realistic and diverse synthetic images, we can augment existing datasets, enhancing the model's training process and improving its performance. This approach can be extended to other medical imaging tasks, offering a scalable solution to the data scarcity problem in medical AI research. The integration of advanced ML techniques in our study represents a significant step forward in AI-driven cancer diagnosis and treatment. The CDCNN model, combined with interpretability and synthetic data generation methods, exemplifies how AI can be harnessed to develop powerful diagnostic tools. These tools not only improve diagnostic accuracy but also provide actionable insights that can guide treatment planning and monitoring. The findings from our study provide significant contributions to the field of medical imaging, offering practical applications that can enhance diagnostic accuracy, support clinical decision-making and improve patient outcomes. By addressing key challenges such as data scarcity and interpretability, our research paves the way for the development of more robust and reliable AI-driven diagnostic tools in healthcare.

### Practical applications

#### Clinical decision support systems (CDSS)

The CDCNN model can be integrated into CDSS to provide radiologists and oncologists with accurate tumour classifications, aiding in diagnosis and treatment planning.

#### Telemedicine

In regions with limited access to expert radiologists, the model can be used in telemedicine applications to provide reliable diagnostic support, ensuring timely and accurate diagnoses for patients in remote areas.

#### Medical education

The synthetic datasets generated by the Pix2Pix GAN model can be used to create extensive training materials for medical students and professionals, offering a wide variety of case studies and imaging conditions.

#### Research and development

The techniques developed in this study can be applied to other types of medical imaging and pathology classification tasks, fostering further research and development in the field of AI-driven medical diagnostics.

#### Limitations and future research directions

The proposed method, despite showing promising results, has several limitations that need to be addressed. Firstly, its ability to generalize to diverse and heterogeneous datasets beyond those from Kaggle and synthetic Pix2Pix GAN data needs further exploration. Additionally, the use of synthetic data may lack real-world nuances, requiring improvements in data quality and the use of varied GAN architectures. Furthermore, the method requires substantial computational resources, highlighting the need for efficiency optimisation. The interpretability of the model remains challenging, necessitating the development of techniques to enhance explainability for clinical trust. It is also important to validate the performance of the model on unseen data to address issues of overfitting and underfitting, which suggests the use of cross-validation and ensemble learning. Lastly, no real-time implementation of the models in clinical environments for system integration was carried out, this must be addressed in future studies.

Subsequent studies could overcome these limitations by incorporating multimodal data, such as merging MRI images with other medical data, to improve the accuracy of diagnosis. This can be achieved by employing sophisticated GAN architectures like diffusion models to generate more realistic synthetic data. Additionally, the development of interpretability techniques to visualize the decision-making process, evaluating the model's performance in different clinical scenarios and brain tumour types, and implementing and testing the model in real-time clinical workflows to assess its feasibility and impact would be beneficial. Tackling these instructions can enhance the proposed approach and potentially propel AI-powered cancer detection and treatment.

## Data Availability

The dataset used in our research work is from the Kaggle dataset, which is openly available for experimentation. The codes we used for this work are openly available at https://github.com/Efep3332/GANs-Generated-Synthetic-Dataset.
